# Potential of an Enzyme Mixture of Glucose Oxidase, Glucosyl Transferase, and Fructosyl Transferase as an Antidiabetic Medicine

**DOI:** 10.3390/biomedicines9070745

**Published:** 2021-06-28

**Authors:** Daham Kim, Juyeon Yu, Eun Kyung Wang, Soohyun Lee, Jung Seung Kim, Jihwan Hwang, Cheol Ryong Ku, Yoon Hee Cho, Eun Jig Lee

**Affiliations:** 1 Department of Internal Medicine, Endocrinology, Institute of Endocrine Research, Yonsei University College of Medicine, Seoul 03722, Korea; kdhs82@yuhs.ac (D.K.); juyeon07@yuhs.ac (J.Y.); ekwang06@yuhs.ac (E.K.W.); golden-ring@nensys.co.kr (J.H.); cr079@yuhs.ac (C.R.K.); wooriminji@gmail.com (Y.H.C.); 2 Department of Internal Medicine, Graduate School of Medical Science, Brain Korea 21 Project, Yonsei University College of Medicine, Seoul 03722, Korea; shlee225@yuhs.ac; 3 NERIG Inc., Seoul 04166, Korea; imkjs77@gmail.com

**Keywords:** glucose oxidase, glucosyl transferase, fructosyl transferase, antidiabetic effect, gut microbiota, lipid profile

## Abstract

An enzyme mixture (EM) of glucose oxidase, glucosyl transferase, and fructosyl transferase can regulate glucose absorption into the body by converting carbohydrates in food to indigestible oligosaccharides. We evaluated the antidiabetic effects of repeated oral administration of EM in db/db mice. Seven-week-old db/db mice were divided into control, voglibose, and EM groups. Drugs were administered orally mixed with limited feed for one month. Glucose levels were measured every week. A meal tolerance test was conducted after overnight fasting, before the mice were sacrificed. There were no differences in body weight or food intake between the groups. EM treatment reduced blood glucose levels compared with those in the control group. Blood glucose levels during the meal tolerance test were significantly lower in the EM group than those in the control group. A significant decrease in triglyceride level and a tendency for decreased low-density lipoprotein were observed in the EM group compared with in the control group. The Bacteroidetes-to-Firmicutes ratio was higher in the EM group than that in the control group. EM may be useful for people at risk of hyperglycemia or diabetes who need to safely regulate their blood glucose levels. EM may also improve lipid and gut microbiota profiles.

## 1. Introduction

Diabetes mellitus (DM) is a chronic metabolic disease associated with public health problems worldwide [1]. According to global estimates, in 2019, the prevalence of DM was 9.3% (463 million), and that of impaired glucose tolerance (IGT) was 7.5% (373.9 million); these are expected to increase to 10.9% (700 million) and 8.6% (548.4 million), respectively, by 2045 [2]. Type 2 DM (T2D), which accounts for approximately 90% of total cases, is characterized by a progressive loss of adequate β-cell insulin secretion, often in the context of insulin resistance [3]. The prevalence of T2D increases proportionally with increasing body mass index [4].

Obesity management has benefits in T2D treatment and delays the progression from prediabetes to T2D [5]. Increased physical activity and fitness combined with calorie restriction and weight loss are important in T2D treatment [6]. However, intensive lifestyle interventions, such as diet and exercise, may be difficult to maintain in the long term and demand great effort from individuals. Metformin is used as the first-line T2D pharmacotherapy in combination with lifestyle modifications, unless there are contraindications [7]. Additional and/or alternative agents (sulfonylureas, thiazolidinediones, α-glucosidase inhibitors, dipeptidyl peptidase-4 inhibitors, and sodium-dependent glucose transporter 2) and injectable medications (insulin and glucagon-like peptide-1) may be considered, as the disease progresses [7]. Complementary and alternative medications may include other therapeutic approaches, although the underlying mechanisms remain to be elucidated [8].

The choice of medication is based on patient clinical characteristics and their preferences. Considerations include their effects on cardiovascular and renal comorbidities, efficacy, hypoglycemia risk, impact on weight, cost, and risks of side effects [7]. Various antihyperglycemic drugs and several weight loss medications lower the risk of diabetes in individuals with prediabetes [9]. However, no pharmacologic agent has been approved by the U.S. Food and Drug Administration (FDA) specifically for diabetes prevention. Additionally, the cost, side effects, and durable efficacy of these drugs require consideration. For example, long-term use of metformin, despite having the strongest evidence, may be associated with biochemical vitamin B12 deficiency, and the possibility of gastrointestinal intolerance and lactic acidosis should be considered [10,11,12].

Transglucosidase (or glucosyl transferase) converts carbohydrates to oligosaccharides, such as panose and iso-malto-oligosaccharide, which are fermented to fecal short-chain fatty acids (SCFAs) by gut bacteria [13]. Oligosaccharides in the alimentary tract modulate gut microbiota composition [13]. The microbiota is implicated in T2D pathogenesis and treatment [8]. Transglucosidase decreases postprandial blood glucose levels in individuals with IGT and T2D [14,15]. This enzyme is currently available in the supplement market and has been acknowledged by the FDA as a new dietary ingredient. Enzyme supplements are generally recognized as safe, although they may interfere with other medications. People are taking more control of their health, and enzymes and other supplements are becoming more popular. Recently, sales of enzyme supplements have been steadily increasing [16].

In this study, we evaluated the antidiabetic effects of repeated oral administration of an enzyme mixture (EM) containing glucose oxidase, glucosyl transferase, and fructosyl transferase in db/db mice. Furthermore, the composition of the gut microbiota was analyzed before and after EM treatment.

## 2. Materials and Methods

### 2.1. Materials and Reagents

**Figure 1:**
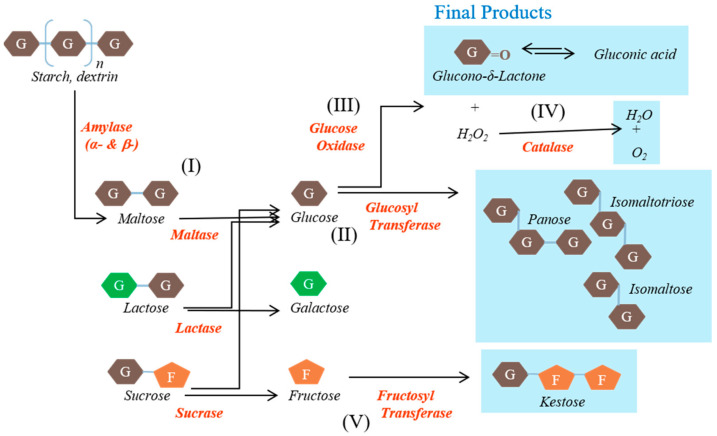
Schematic representation of the activities of the enzymes contained in the enzyme mixture evaluated in this study. Carbohydrates are converted to indigestible oligosaccharides by the various enzymes.

The EM supplement included glucose oxidase, glucosyl transferase, fructosyl transferase, catalase, amylase, and lactase and was provided by NERIG Inc. (Seoul, Korea). The schematic representation of the activities of the enzymes contained in EM is shown in Figure 1.

The main substances, namely glucose oxidase, glucosyl transferase, and fructosyl transferase, play major roles in the generation of unabsorbed sugars. Glucose oxidase converts glucose into organic acids. Glucosyl transferase converts maltose into gluco-oligosaccharides that humans cannot digest, such as isomaltose. Fructosyl transferase converts sucrose into fructo-oligosaccharides that humans cannot use, such as ketoses. The other substances were included to create conditions in which these reactions can occur rapidly or to reduce the likelihood of side effects. Catalase converts hydrogen peroxide, a by-product, into water and oxygen. Amylase converts starch into saccharides such as maltose. Lactase converts lactose into glucose and galactose. The origins and activities of the enzymes contained in the EM are shown in Table 1.

Enzyme activities and dosage were determined through preliminary experiments in vitro and in vivo. The α-glucosidase inhibitor voglibose was purchased from Sigma-Aldrich (St. Louis, MO, USA). Vanilla Ensure Plus was purchased from Abbott Laboratories (Columbus, OH, USA).

### 2.2. Animals and Experimental Design

Animal experiments were approved by the ChemOn Inc. Animal Experiment Ethics Committee (Suwon, Korea) (protocol number: 2020-08-003). Six-week-old male db/db mice (C57BLKS/J lar^–^ Lepr^db^/Lepr^db^) were purchased from Central Laboratory Animal Inc. (Seoul, Korea) and were housed one per cage with a 12-h light/dark cycle at 23 ± 3 °C and a relative humidity of 55% ± 15%. They were maintained on PicoLab Rodent Diet 20 5053 (LabDiet, Columbia, MO, USA) and given free access to water. They were acclimatized for 1 week prior to the study.

The mice were randomized into three groups (*n* = 7 in each group) on the basis of body weight and blood glucose levels assessed 1 day prior to initiating the experiment. Voglibose (0.3 mg/kg/day) or EM (300 mg/kg/day) was mixed with powdered feed, and limited feeding was performed using a mouse feeder shield (approximately 5 g/day, based on a body weight of 33 g) for 1 month. The control group was only fed a limited amount of powdered feed. Food intake was evaluated daily by measuring the remaining amount in the mouse feeder shield [17]. Blood glucose was measured once a week, 4 h after fasting and 2 h after feeding, using a blood glucose meter (AGM-4000; Allmedicus, Anyang, Korea). Body weight was measured twice a week. The meal tolerance test (MTT) was performed after approximately 16 h of fasting on the 28th day of administration. Immediately after the administration of the daily amount of drug dissolved in normal saline, Vanilla Ensure Plus (0.21 g carbohydrate (CHO)/mL: 0.086 g/mL sugar and 0.128 g/mL maltodextrin) was administered by oral gavage at a dose of 1.2 g CHO/kg body weight. The blood was sampled via tail nick at 0, 15, 30, 60, 90, and 120 min post-gavage and immediately analyzed for glucose levels.

### 2.3. Biochemical and Histopathological Examinations

Autopsy was performed 5 days after the MTT. Fasting blood glucose was measured at autopsy after 12 h of fasting as follows. Autopsied animals were subjected to inhalation anesthesia with isoflurane, and then, the blood was collected from the posterior vena cava. After blood collection, the abdominal aorta and posterior vena cava were cut, and the animals were bled out. The collected blood was placed in a vacutainer tube containing a clot activator, left at room temperature for at least 30 min and then centrifuged at 3000 rpm for 10 min. The obtained serum was transferred to a new tube and stored at −80 °C, until biochemical analysis was performed. The liver, heart, spleen, stomach, and kidney were removed and fixed in 10% neutral buffered formalin solution.

Total cholesterol, high-density lipoprotein (HDL), low-density lipoprotein (LDL), triglycerides, aspartate aminotransferase (AST), alanine aminotransferase (ALT), and creatinine were measured using a blood biochemical analyzer (AU680; Beckman Coulter, Mishima, Japan). The fixed tissues were stained with hematoxylin and eosin for histopathological examination. Paraffin blocks were cut into 3–4-μm sections using a microtome (RM2255; Leica Biosystems, Nussloch, Germany), and the sections were examined using a fluorescence microscope (Eclipse 80i; Nikon, Kawasaki, Japan).

### 2.4. Gut Microbiota Analysis

Before the first administration of drugs and the day before autopsy, fresh fecal samples were collected from individual animals and immediately stored in liquid nitrogen until further processing. DNA was extracted from samples using a PowerSoil^®^ DNA Isolation Kit (MO BIO Laboratories, Carlsbad, CA, USA). After performing quality control, amplification, sequencing, and library preparation were conducted using a Herculase II fusion DNA polymerase Nextera XT Index Kit V2, following the 16S metagenomic sequencing library preparation (Part #15044223 Rev. B) protocol on an Illumina platform at Macrogen (Seoul, Korea) and yielding paired end reads.

### 2.5. Statistical Analysis

IBM SPSS Statistics version 25 (IBM, Armonk, NY, USA) was used for all statistical analyses. Data are presented as the mean ± standard error of the mean (SEM). Statistical significance was determined using the Student’s *t*-test. Comparisons between the control and the voglibose or EM groups were performed. *p* of <0.05 indicated statistical significance.

## 3. Results

### 3.1. Antidiabetic Effects of EM Administration

**Figure 2:**
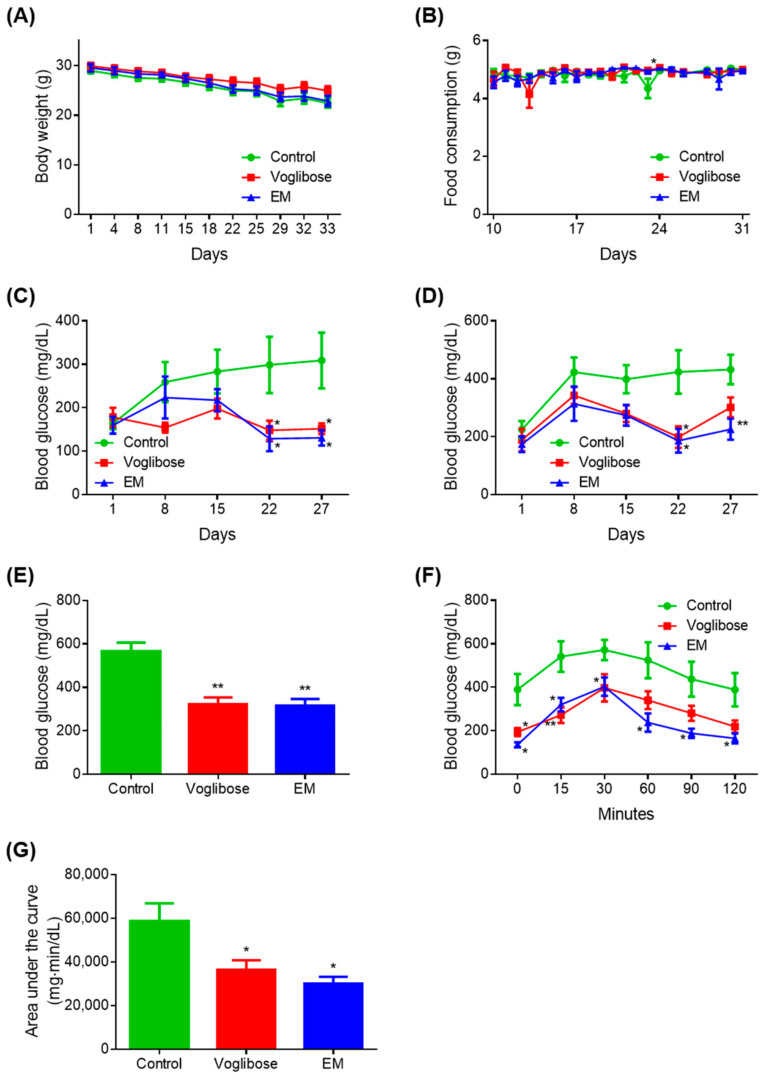
Antidiabetic effects of the enzyme mixture administration. (**A**) Body weight; (**B**) food intake; (**C**) blood glucose 4 h after fasting; (**D**) blood glucose 2 h after feeding; (**E**) blood glucose at autopsy 12 h after fasting; (**F**) blood glucose during the meal tolerance test; and (**G**) glucose area under the curve during the meal tolerance test. Data are expressed as the mean ± standard error of the mean (SEM; *n* = 7). * *p* < 0.05 and ** *p* < 0.01 vs. control (Student’s *t*-test).

There was no difference in body weight in the voglibose and EM groups compared with the control group (Figure 2A). The voglibose group showed a significant increase in food intake on day 24 compared with the control group (*p* < 0.05); however, there was no difference at other measured time points (Figure 2B). There was no difference in food intake in the EM group compared with in the control group.

A significant decrease in blood glucose was observed on days 22 and 27 in the voglibose and EM groups compared with in the control group, when blood glucose was measured after fasting for 4 h (*p* < 0.050) (Figure 2C). The voglibose group showed a significant decrease in blood glucose on day 22 (*p* < 0.050) and a tendency for decreased blood glucose on days 15 and 27 when blood glucose was measured 2 h after feeding (*p* = 0.055) (Figure 2D).

Compared with the control group, the EM group showed significant decreases in blood glucose on days 22 and 27 (*p* < 0.050 or *p* < 0.010), and a decreasing trend was observed on day 15 (*p* = 0.060). There was no difference between the voglibose and EM groups compared with the control group at any other time point measured. The voglibose and EM groups showed significant decreases in blood glucose compared with the control group, when blood glucose was measured at autopsy after 12 h of fasting (*p* < 0.010) (Figure 2E).

In the MTT, the voglibose group showed a significant decrease in blood glucose at 0, 15, and 30 min compared with the control group (*p* < 0.050 or *p* < 0.010), and 35.14%, 35.89%, and 43.68% tendencies for decreased blood glucose were observed at 60, 90, and 120 min, respectively (Figure 2F,G). In the EM group, significant decreases in blood glucose and the area under the curve were observed compared with those in the control group (*p* < 0.050).

### 3.2. Biochemical and Histopathological Examination after EM Administration

**Figure A1:**
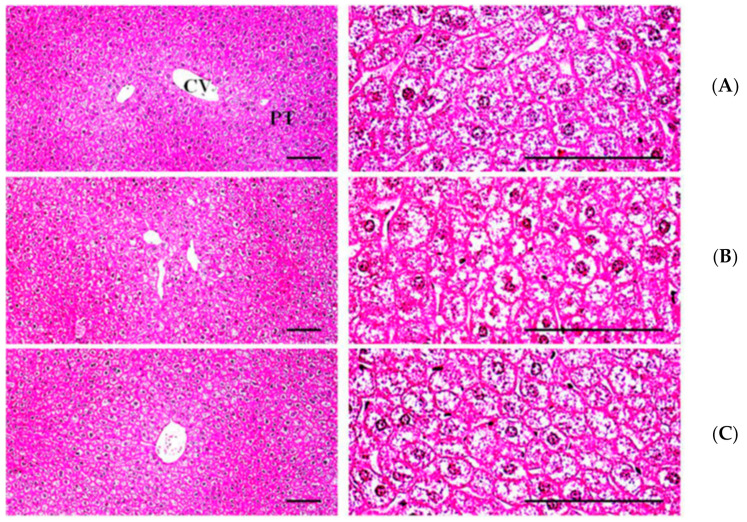
Representative histopathological profiles of liver tissues: (**A**) normal diet control (*n* = 7); (**B**) voglibose (*n* = 7); (**C**) enzyme mixture (*n* = 7). CV, central vein; PT, portal triad. Scale bars: 100 μm.

**Figure A2:**
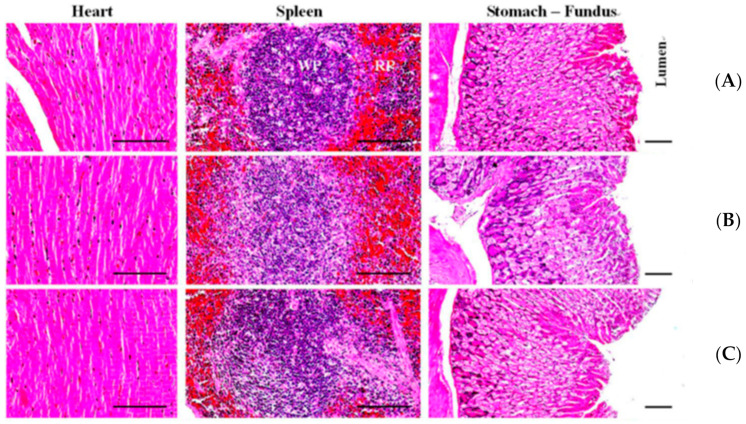
Representative histopathological profiles of the heart, spleen, and stomach tissues: (**A**) normal diet control (*n* = 7); (**B**) voglibose (*n* = 7); (**C**) enzyme mixture (*n* = 7). WP, white pulp; RP, red pulp. Scale bars: 100 μm.

**Figure A3:**
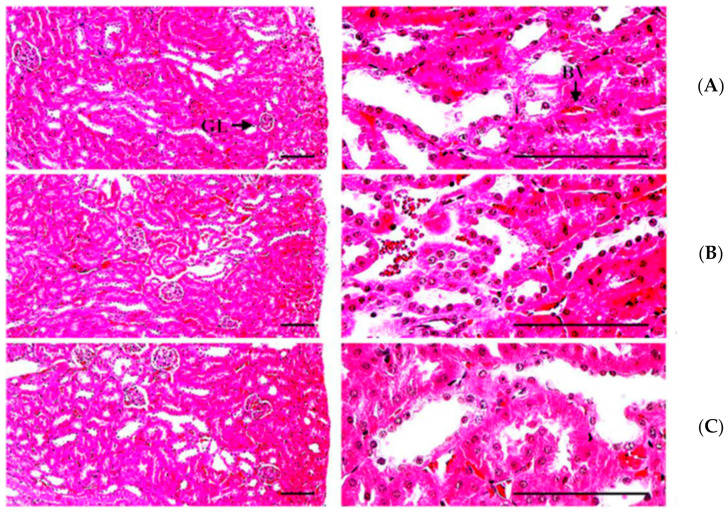
Representative histopathological profiles of kidney tissues: (**A**) normal diet control (*n* = 7); (**B**) voglibose (*n* = 7); (**C**) enzyme mixture (*n* = 7). GL, glomerulus; BV, blood vessel. Scale bars: 100 μm.

There were no differences in HDL, AST, ALT, and creatinine levels in the voglibose and EM groups compared with those in the control group; however, significant decreases in total cholesterol of the voglibose group and in triglyceride level of the voglibose and EM groups were observed (*p* < 0.05) (Table 2). There was no significant difference in LDL, but decreases of 36.26% and 32.60% were observed in the voglibose and EM groups, respectively. There was no difference in the histopathological examination results, such as steatohepatitis regions, degenerative tubule numbers, mucosa thicknesses, in the voglibose and EM groups compared with in the control group. Representative images are shown in Figure A1, Figure A2 and Figure A3. The EM supplementation did not have any negative side effects in the mice.

### 3.3. Gut Microbiota Analysis before and after EM Administration

**Figure 3:**
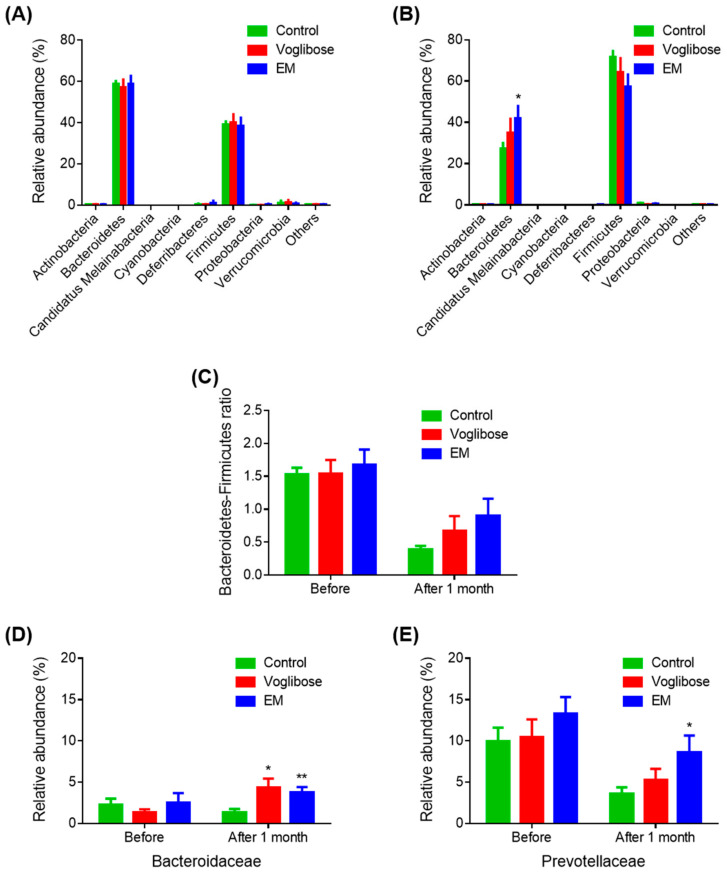
Gut microbiota analysis before and after enzyme mixture administration. Gut microbiota composition before (**A**) and after (**B**) drug administration at the phylum level; (**C**) Bacteroidetes-to-Firmicutes ratios; and (**D**,**E**) relative abundances of the Bacteroidaceae (**D**) and Prevotellaceae (**E**) families. Data are expressed as the mean ± SEM, (*n* = 7). * *p* < 0.05 and ** *p* < 0.01 vs. control (Student’s *t*-test).

To investigate the effects of EM on gut bacterial composition, the gut microbiota compositions before and after drug administration in the control, voglibose, and EM groups were analyzed. Bacteroidetes was the most abundant phylum (57.11%–58.75%), and Firmicutes was the second most abundant phylum (38.30%–40.17%) in all groups before drug administration (Figure 3A). After 1 month, on the day before autopsy, Firmicutes was the most abundant phylum (57.13%–71.70%), and Bacteroidetes was the second most abundant phylum (27.27%–42.00%) in all groups (Figure 3B). The EM group had a significantly high abundance of Bacteroidetes compared with the control group (*p* = 0.044). Before treatment, no difference was observed in the Bacteroidetes-to-Firmicutes ratio among the three groups (Figure 3C). After 1 month of treatment, the Bacteroidetes-to-Firmicutes ratio decreased in all groups. However, the Bacteroidetes-to-Firmicutes ratio in the EM group was higher than that in the control group, indicating improvement in the gut microbiota, although the difference was not significant (*p* = 0.082).

Next, family-level results were examined (Figure 3D,E). The abundances of Bacteroidaceae were significantly increased in the voglibose and EM groups compared with those in the control group (*p* = 0.021 and *p* = 0.009, respectively). The abundance of Prevotellaceae in the EM group was significantly higher than that in the control group after one month of treatment (*p* = 0.039). Other families were not changed in the voglibose and EM groups compared with those in the control group. The relative increase in the phylum Bacteroidetes in the EM group was because of the increase in the microbial populations of the Bacteroidaceae and Prevotellaceae families. Considering the EM mechanism, EM indirectly affects the gut microbiota via a higher input of oligosaccharides in the gut.

## 4. Discussion

T2D is a metabolic disorder characterized by obesity-related insulin resistance [18]. While carbohydrate restriction may help maintain weight loss and maximize metabolic benefits, it is difficult to maintain and demand great effort; enzymes such as transglucosidase, which regulates glucose absorption, are relatively safe and may achieve the same results [14,15,19]. In this study, the antidiabetic effects of repeated oral administration of EM, an enzyme supplement aimed at regulating sugar metabolism, were evaluated in db/db mice. As expected, mice treated with EM showed lower blood glucose levels without any negative side effects, as determined by biochemical and histopathological examinations. Notably, EM administration improved the lipid and gut microbiota profiles.

In a preliminary study in which EM was administered orally twice daily for one month, the blood-glucose-lowering effect was not as strong as expected, likely because db/db mice exhibited severe weight gain and increased blood glucose levels as they aged, and EM should be administered with a diet to be effective [20]. Therefore, in this study, the drugs were mixed with a limited amount of feed. Based on the feed intake in the preliminary study, the amount of feed given was 5 g/day, based on a body weight of 33 g. This is approximately 80% of the minimum amount per day and considers the possibility of loss and the minimum amount eaten by the mice. Thus, it was hypothesized that the feed would be entirely consumed. There was no difference in feed intake among the three groups; however, the mouse feeder shield was not well used at the beginning of the experiment, and thus, the dietary intake was not accurately measured at that time [17]. Body weights tended to slightly decrease in all three groups, which is likely related to the limited feed [20]. Theoretically, both the voglibose and EM groups inhibited glucose uptake; however, there was no difference in body weight compared with that in the control group during the study period.

The blood-glucose-lowering effect before and after feeding was more pronounced than that in the preliminary study, suggesting that the treatment was effective because the drug was administered with the feed. Additionally, the limited feed per se was thought to have contributed to the effect by mitigating the continuous rise in blood glucose as the mice aged [20]. Because T2D was improved by continuous administration of EM, blood glucose during the MTT and fasting blood glucose at autopsy also showed significant differences compared with those in the control group. Based on this study, EM is expected to exhibit strong antidiabetic effects when administered with the diet.

Along with lowered blood glucose, the lipid profile, especially the level of triglycerides, was significantly improved. When the content of dietary carbohydrate was high, blood triglyceride levels increased [21]. It seemed that EM reduced the triglyceride levels, because it reduced the amount of carbohydrates ingested. Reduction in the consumption of added sugars, particularly added fructose, may translate into reduced diabetes-related morbidity and premature mortality [22]. EM contains not only glucose oxidase and glucosyl transferase, which reduce glucose, but also fructosyl transferase, which reduces fructose. This may have a similar effect to that by reducing the intake of added sugars.

In the stomach, EM converts carbohydrates in food to indigestible oligosaccharides, which are then decomposed into glucose and absorbed in the small intestine by the activity of various enzymes, such as maltase, sucrase, and lactase (WO2019093663A1). By this mechanism, carbohydrates should be rapidly broken down into monosaccharides before they reach the small intestine, and the added amylase and lactase effectively help with this. All EM components are natural enzymes, and there is little possibility of side effects. However, hydrogen peroxide, a by-product of glucose oxidase, may cause irritation at high concentrations; therefore, catalase is added to prevent irritation [23,24]. Nevertheless, previous studies have reported that glucose oxidase affects the intestinal environment by utilizing oxygen to produce gluconic acid and hydrogen peroxide, which are toxic to pathogenic bacteria but promote the survival of beneficial bacteria [25]. Reactive oxygen species, such as hydrogen peroxide, are essential chemicals for resolving infectious disease and are currently recognized to modify the microbiota composition and hence, improve colonization resistance [26]. Cell-based studies have shown that hydrogen peroxide exhibits a pathogen-repellent effect that reduces bacterial invasion [27,28]. An in vitro study demonstrated that EM specifically inhibits the growth of *Salmonella typhimurium* without inhibiting the growth of *Bacillus subtilis*, *Escherichia coli*, and *Lactobacillus acidophilus* (unpublished). *Lactobacilli* are beneficial probiotics, and their beneficial effects are linked to hydrogen peroxide, one of the factors they secrete [29,30,31]. However, the abundance of *Lactobacilli* did not differ between three groups in this study.

Indigestible oligosaccharides help to regulate blood glucose and insulin levels, promote health and prevent diseases such as T2D [32]. Dietary fiber that includes oligosaccharides has important beneficial effects on the microbiota in the large intestine [14,33,34]. EM converts carbohydrates to indigestible oligosaccharides and modulates the gut microbiota composition. In gut microbiota analysis in this study, the Bacteroidetes-to-Firmicutes ratio was flipped, possibly because obesity is linked to alterations in the intestinal microbiota, although a slight loss of body weight was achieved with the limited feed [35,36,37]. The EM group showed a significantly high abundance of Bacteroidetes and a relatively high Bacteroidetes-to-Firmicutes ratio compared with the control group after one month of treatment. These findings are consistent with those in a previous study showing that the relative proportion of Bacteroidetes in obese people is decreased compared with that in lean people but increases with weight loss on a low-energy diet [37]. Treatment with transglucosidase, a major component of EM, significantly increases the Bacteroidetes-to-Firmicutes ratio, indicating that transglucosidase improves the growth of gut bacterial communities in T2D patients [13]. The relative increase in the abundance of Bacteroidetes in the EM group was mainly because of the increases in the abundances of Bacteroidaceae and Prevotellaceae. Prebiotic-xylooligosaccharide-fed mice have a higher abundance of *Prevotella* species in their gut microbiota than in control mice [38]. In response to acarbose, an increase in the number of SCFA-producing taxa, such as *Faecalibacterium*, *Prevotella*, and *Lactobacillus*, has been observed [39]. In this study, the EM group rather than the voglibose group showed a relatively high abundance of *Prevotella*, a genus in the family *Prevotellaceae*, compared with the control group. However, the effects of EM on the gut microbiota remain uncertain, necessitating further studies.

EM can be used, regardless of liver or kidney function, because it converts carbohydrates to indigestible oligosaccharides. Additionally, EM may be taken in combination with most antidiabetic medications; however, it would be better not to use it with α-glucosidase inhibitors. In contrast to EM, α-glucosidase inhibitors delay the absorption of carbohydrates from the small intestine, acting as competitive inhibitors of enzymes needed to digest carbohydrates, and thus have a lowering effect on blood glucose and insulin levels after a meal [40]. EM and α-glucosidase inhibitors can be considered as similar classes, in that they reduce the impact of dietary carbohydrates on blood glucose. Therefore, the voglibose was used as a positive control in this study.

The strengths of this study are that the EM effect was confirmed with relatively long-term administration rather than a single administration and the effect was clearly confirmed upon administration with feed, which is consistent with the mechanism of EM. The limitations of this study are that the study period was insufficiently long to demonstrate the expected weight loss, the possibility that the limited feed was exceeded, and that the mouse feeder shield was not well used at the beginning of the experiment, and therefore, dietary intake could not be accurately measured at that time. However, later in the experiment, dietary intake was measured aptly, there was no difference in dietary intake among the groups, and EM showed antidiabetic effects without any side effects.

In conclusion, EM may be useful for people at risk of hyperglycemia or diabetes who need to safely regulate their blood glucose levels. Additionally, EM may improve lipid and gut microbiota profiles.

## Figures and Tables

**Table 1:** The origins and activities of the enzymes contained in the enzyme mixture.

Enzyme	Reaction Step(s)	Origin	Activity (U/mg)
Glucose oxidase	(III)	*Aspergillus niger*	25
Glucosyl transferase	(I) and (II)	*Aspergillus niger*	7.5
Fructosyl transferase	(I) and (V)	*Aspergillus niger*	0.25
Catalase	(IV)	*Aspergillus niger*	125
Amylase	(I)	*Aspergillus oryzae*	1.25
Lactase	(I)	*Aspergillus niger*	1

**Table 2:** Biochemical examination after EM administration.

Biochemical Analyte	Control	Voglibose	EM
Total cholesterol (mg/dL)	83.00 ± 8.21	58.29 ± 3.06 *	75.29 ± 9.91
High-density lipoprotein (mg/dL)	52.63 ± 5.38	43.46 ± 2.63	54.24 ± 7.62
Low-density lipoprotein (mg/dL)	19.50 ± 3.61	12.43 ± 0.78	13.14 ± 1.20
Triglycerides (mg/dL)	124.50 ± 13.76	88.29 ± 6.64 *	91.14 ± 6.34 *
Aspartate aminotransferase (U/L)	80.80 ± 6.83	82.41 ± 7.81	89.70 ± 9.75
Alanine aminotransferase (U/L)	49.97 ± 4.57	57.56 ± 4.19	61.10 ± 7.22
Creatinine (mg/dL)	0.29 ± 0.01	0.25 ± 0.03	0.28 ± 0.01

Abbreviation: EM, enzyme mixture. Data are expressed as the mean ± SEM (*n* = 7). * *p* < 0.05 vs. control (Student’s *t*-test).

## Data Availability

Data can be available upon request.

## References

[1] Al-Lawati J.A. (2017). Diabetes Mellitus: A Local and Global Public Health Emergency!. Oman Med. J..

[2] Saeedi P., Petersohn I., Salpea P., Malanda B., Karuranga S., Unwin N., Colagiuri S., Guariguata L., Motala A.A., Ogurtsova K. (2019). Global and regional diabetes prevalence estimates for 2019 and projections for 2030 and 2045: Results from the International Diabetes Federation Diabetes Atlas, 9th edition. Diabetes Res. Clin. Pract..

[3] American Diabetes Association (2021). 2. Classification and Diagnosis of Diabetes: Standards of Medical Care in Diabetes—2021. Diabetes Care.

[4] Willett W.C., Dietz W.H., Colditz G. (1999). Guidelines for Healthy Weight. N. Engl. J. Med..

[5] American Diabetes Association (2021). 8. Obesity Management for the Treatment of Type 2 Diabetes:Standards of Medical Care in Diabetes—2021. Diabetes Care.

[6] Magkos F., Hjorth M.F., Astrup A. (2020). Diet and exercise in the prevention and treatment of type 2 diabetes mellitus. Nat. Rev. Endocrinol..

[7] American Diabetes Association (2021). 9. Pharmacologic Approaches to Glycemic Treatment: Standards of Medical Care in Diabetes—2021. Diabetes Care.

[8] Zheng Y., Gou X., Zhang L., Gao H., Wei Y., Yu X., Pang B., Tian J., Tong X., Li M. (2020). Interactions Between Gut Microbiota, Host, and Herbal Medicines: A Review of New Insights into the Pathogenesis and Treatment of Type 2 Diabetes. Front. Cell Infect. Microbiol..

[9] American Diabetes Association (2021). 3. Prevention or Delay of Type 2 Diabetes: Standards of Medical Care in Diabetes—2021. Diabetes Care.

[10] Knowler W.C., Barrett-Connor E., Fowler S.E., Hamman R.F., Lachin J.M., Walker E.A., Nathan D.M. (2002). Reduction in the Incidence of Type 2 Diabetes with Lifestyle Intervention or Metformin. N. Engl. J. Med..

[11] The Diabetes Prevention Program Research Group (2012). Long-Term Safety, Tolerability, and Weight Loss Associated with Metformin in the Diabetes Prevention Program Outcomes Study. Diabetes Care.

[12] Aroda V.R., Edelstein S.L., Goldberg R.B., Knowler W.C., Marcovina S.M., Orchard T., Bray G.A., Schade D.S., Temprosa M.G., White N.H. (2016). Long-term Metformin Use and Vitamin B12 Deficiency in the Diabetes Prevention Program Outcomes Study. J. Clin. Endocrinol. Metab..

[13] Sasaki M., Ogasawara N., Funaki Y., Mizuno M., Iida A., Goto C., Koikeda S., Kasugai K., Joh T. (2013). Transglucosidase improves the gut microbiota profile of type 2 diabetes mellitus patients: A randomized double-blind, placebo-controlled study. BMC Gastroenterol..

[14] Sasaki M., Joh T., Koikeda S., Kataoka H., Tanida S., Oshima T., Ogasawara N., Ohara H., Nakao H., Kamiya T. (2007). A Novel Strategy in Production of Oligosaccharides in Digestive Tract: Prevention of Postprandial Hyperglycemia and Hyperinsulinemia. J. Clin. Biochem. Nutr..

[15] Sasaki M., Imaeda K., Okayama N., Mizuno T., Kataoka H., Kamiya T., Kubota E., Ogasawara N., Funaki Y., Mizuno M. (2011). Effects of transglucosidase on diabetes, cardiovascular risk factors and hepatic biomarkers in patients with type 2 diabetes: A 12-week, randomized, double-blind, placebo-controlled trial. Diabetes Obes. Metab..

[16] Daliri E.B.-M., Lee B.H. (2015). Current Trends and Future Perspectives on Functional Foods and Nutraceuticals. Beneficial Microorganisms in Food and Nutraceuticals.

[17] Rehrig A., DeMagistris M., Callan C. (2013). Refinements in laboratory cat management: While the humans are away, the cats will play!. Lab. Anim. Sci. Prof..

[18] Czech M.P. (2017). Insulin action and resistance in obesity and type 2 diabetes. Nat. Med..

[19] Hyde P.N., Sapper T.N., Crabtree C.D., LaFountain R.A., Bowling M.L., Buga A., Fell B., McSwiney F., Dickerson R.M., Miller V.J. (2019). Dietary carbohydrate restriction improves metabolic syndrome independent of weight loss. JCI Insight.

[20] Hummel K.P., Dickie M.M., Coleman D.L. (1966). Diabetes, a New Mutafton in the Mouse. Science.

[21] Parks E.J. (2001). Effect of Dietary Carbohydrate on Triglyceride Metabolism in Humans. J. Nutr..

[22] DiNicolantonio J.J., O’Keefe J.H., Lucan S.C. (2015). Added Fructose. Mayo Clin. Proc..

[23] Bankar S.B., Bule M.V., Singhal R., Ananthanarayan L. (2009). Glucose oxidase—An overview. Biotechnol. Adv..

[24] Kapat A., Jung J.-K., Park Y.-H. (1998). Improvement of extracellular recombinant glucose oxidase production in fed-batch culture of Saccharomyces cerevisiae: Effect of different feeding strategies. Biotechnol. Lett..

[25] Wu S., Li T., Niu H., Zhu Y., Liu Y., Duan Y., Sun Q., Yang X. (2019). Effects of glucose oxidase on growth performance, gut function, and cecal microbiota of broiler chickens. Poult. Sci..

[26] Knaus U.G., Hertzberger R., Pircalabioru G.G., Yousefi S.P.M., Dos Santos F.B. (2016). Pathogen control at the intestinal mucosa—H_2_O_2_ to the rescue. Gut Microbes.

[27] Botteaux A., Hoste C., Dumont J., Van Sande J., Allaoui A. (2009). Potential role of Noxes in the protection of mucosae: H_2_O_2_ as abacterial repellent. Microbes Infect..

[28] Corcionivoschi N., Alvarez L.A., Sharp T., Strengert M., Alemka A., Mantell J., Verkade P., Knaus U.G., Bourke B. (2012). Mucosal Reactive Oxygen Species Decrease Virulence by Disrupting Campylobacter jejuni Phosphotyrosine Signaling. Cell Host Microbe.

[29] van Baarlen P., Wells J.M., Kleerebezem M. (2013). Regulation of intestinal homeostasis and immunity with probiotic lactobacilli. Trends Immunol..

[30] Saez-Lara M.J., Gomez-Llorente C., Plaza-Diaz J., Gil A. (2015). The Role of Probiotic Lactic Acid Bacteria and Bifidobacteria in the Prevention and Treatment of Inflammatory Bowel Disease and Other Related Diseases: A Systematic Review of Randomized Human Clinical Trials. BioMed Res. Int..

[31] Wasilewski A., Zielińska M., Storr M., Fichna J. (2015). Beneficial Effects of Probiotics, Prebiotics, Synbiotics, and Psychobiotics in Inflammatory Bowel Disease. Inflamm. Bowel Dis..

[32] Sako T., Mori A., Lee P., Goto H., Fukuta H., Oda H., Saeki K., Miki Y., Makino Y., Ishioka K. (2010). Supplementing transglucosidase with a high-fiber diet for prevention of postprandial hyperglycemia in streptozotocin-induced diabetic dogs. Vet. Res. Commun..

[33] Gibson G.R. (1999). Dietary Modulation of the Human Gut Microflora Using the Prebiotics Oligofructose and Inulin. J. Nutr..

[34] Langlands S.J., Hopkins M.J., Coleman N., Cummings J.H. (2004). Prebiotic carbohydrates modify the mucosa associated microflora of the human large bowel. Gut.

[35] Ley R.E., Bäckhed F., Turnbaugh P., Lozupone C.A., Knight R.D., Gordon J.I. (2005). Obesity alters gut microbial ecology. Proc. Natl. Acad. Sci. USA.

[36] Turnbaugh P.J., Bäckhed F., Fulton L., Gordon J.I. (2008). Diet-Induced Obesity Is Linked to Marked but Reversible Alterations in the Mouse Distal Gut Microbiome. Cell Host Microbe.

[37] Ley R., Turnbaugh P.J., Klein S., Gordon J.I. (2006). Human gut microbes associated with obesity. Nat. Cell Biol..

[38] Laigaard A., Krych L., Zachariassen L.F., Ellegaard-Jensen L., Nielsen D.S., Hansen A.K., Hansen C.H.F. (2020). Dietary prebiotics promote intestinal Prevotella in association with a low-responding phenotype in a murine oxazolone-induced model of atopic dermatitis. Sci. Rep..

[39] Zhang X., Fang Z., Zhang C., Xia H., Jie Z., Han X., Chen Y., Ji L. (2017). Effects of Acarbose on the Gut Microbiota of Prediabetic Patients: A Randomized, Double-blind, Controlled Crossover Trial. Diabetes Ther..

[40] Van De Laar F.A., Lucassen P.L., Akkermans R.P., Van De Lisdonk E.H., Rutten G.E., Van Weel C. (2004). Glucosidase Inhibitors for Patients with Type 2 Diabetes: Results from a Cochrane systematic review and meta-analysis. Diabetes Care.

